# Synthesis, Characterization and Antiproliferative Activity of the Co(II), Ni(II), Cu(II), Pd(II) and Pt(II) Complexes of 2-(4-Thiazolyl)Benzimidazole (Thiabendazole)

**DOI:** 10.1155/MBD.2001.189

**Published:** 2001

**Authors:** Marek Z. Wisniewski, Tadeusz Glowiak, Adam Opolski, Joanna Wietrzyk

**Affiliations:** 1 Institute of Chemistry Swietokrzyski University Checinska 5 Kielce 25-020 Poland; 2 Institute of Chemistry University of Wroclaw F. Joliot-Curie 40 Wroclaw 50-383 Poland; 3 Institute of Immunology and Experimental Therapy Polish Academy of Sciences Weigla 12 Wroclaw 53-114 Poland

## Abstract

Complexes of 2-(4-thiazolyi)benzimidazole (thiabendazole, THBD) with Co(II), Ni(II), Cu(ll) of general formula ML_2_(NO_3_)_2_ H_2_O and complexes of Pd(II) and Pt(II) of general formula ML2Cl_2_ H_2_O have been obtained and characterized by elemental analyses, IR and far IR spectroscopy and magnetic measurements. The X-ray crystal structure of the copper(II) complex has been determined. The *in vitro* cell proliferation inhibitory activity of these compounds was examined against human cancer cell lines A 549 (lung carcinoma), HCV-29 T (urinary bladder carcinoma), MCF-7 (breast cancer), T47D (breast cancer), MES-SA (uterine carcinoma) and HL-60 (promyelocytic leukemia). Pt-THBD has been found to exhibit an antileukemic activity of the HL-60 line cells matching that of an arbitrary criterion.


### SYNTHESIS, CHARACTERIZATION AND ANTIPROLIFERATIVE ACTIVITY OF THE Co(II), Ni(II), Cu(II), Pd(II) AND Pt(II) COMPLEXES OF 2-(4-THIAZOLYL)BENZIMIDAZOLE (THIABENDAZOLE)

Marek Z. Wisniewski*[1], Tadeusz Glowiak[2], Adam Opolski[3], and Joanna Wietrzyk[3]

[1] Institute of Chemistry, Swietokrzyski University, Checinska 5, 25-020 Kielce, Poland
<wisem@pu.kielce.pl>
[2] Institute of Chemistry, University of Wroclaw, F. Joliot-Curie 40, 50-383 Wroclaw, Poland
[3] Institute of Immunology and Experimental Therapy, Polish Academy of Sciences, Weigla 12, 53-114 Wroclaw, Poland

### ABSTRACT

Complexes of 2-(4-thiazolyl)benzimidazole (thiabendazole, THBD) with Co(II), Ni(II), Cu(II) of general formula $ML_2(NO_3)_2\ H_2O$ and complexes of Pd(II) and Pt(II) of general formula $ML_2Cl_2\ H_2O$ have been obtained and characterized by elemental analyses, IR and far IR spectroscopy and magnetic measurements. The X-ray crystal structure of the copper(II) complex has been determined. The *in vitro* cell proliferation inhibitory activity of these compounds was examined against human cancer cell lines A 549 (lung carcinoma), HCV-29 T (urinary bladder carcinoma), MCF-7 (breast cancer), T47D (breast cancer), MES-SA (uterine carcinoma) and HL-60 (promyelocytic leukemia). Pt-THBD has been found to exhibit an antileukemic activity of the HL-60 line cells matching that of an arbitrary criterion.

### INTRODUCTION

Co-ordination compounds of metals have found increasing application for the treatment of many diseases. For instance, they have been used as haematopoietic, antiphlogistic, bactericidal, antiarthiric and antitumor drugs [1-3]. Studies of antitumor properties of platinum complexes have been triggered by the detection by Rosenberg [4,5] of selective inhibition of cell division by *cis*- $Pt(NH_3)_2Cl_2$ and *cis*- $Pt(NH_3)_2Cl_4$ complexes. Their antitumor activity rests on the inhibition of DNA synthesis in the tumor cell, and the structure of a compound being formed resembles that of cross-linked chains [6,7].

By now, platinum a containing antitumor drugs of a new generation, carboplatin, iproplatin and spiroplatin, have gained widespread application [8]. Recently, studies of antitumor properties of the complexes of Ni(II), Mn(II), Co(II), Cu(II), Zn(II), Ru(II), Sn(II), Zr(II) and Ti(II) have been reported [9-11].

Many research workers have also studied the antitumor properties of complexes with azole ligands [12-16]. In this connection it seemed worthwhile to study the activity of complexes of Co(II), Ni(II), Cu(II), Pd(II) and Pt(II) with a popular antiparasitic drug, 2-(4-thiazolyl)benzimidazole (thiabendazole, THBD; Fig.1). The distribution of charge densities in the ligands was calculated by PM3 semiempirical methods using a Hyper Chem 5.01 computer program for Windows 95 from Hypercube Inc. The purpose of this contribution was to obtain an information about the composition of the complexes, co-ordination arrangement of the central ion and to identify electron-donating atoms of the ligand as well as to determine the antiproliferative activity of the complexes against cells of selected human tumor lines.

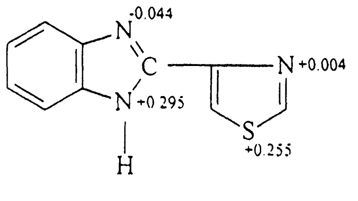

Fig.1. The charge density distribution of THBD

### MATERIALS AND METHODS

### Reagents

2-(4-thiazolyl)benzimidazole (thiabendazole, THBD) was purchased from Sigma. The cobalt(II), nickel(II) and copper(II) nitrates (POCh, Gliwice, Poland) were crystallized from redistilled water. The preparation of $PdCl_2$ and $K_2PdCl_4$ was described previously [17].

### Physical measurements

Magnetic measurements at ambient temperature were run on a MSB-MKI (Sherwood Scientific Ltd.) instrument. Elemental analyses were run on a Model 240 Perkin-Elmer CHN Analyzer.

The IR spectra (4000–250 $cm^{-1}$) were taken on a Beckmann Model 4240 spectrophotometer in KBr discs. The far-IR spectra (450–80 $cm^{-1}$) were recorded on a DiGiLab FTS-60 spectrophotometer by applying suspensions of the compounds in acetone onto the polyethylene window. Melting points were determined on a Boetius apparatus. Magnetic measurements were accomplished on a MSB-MKI Instrument (Sherwood Scientific Ltd) at ambient temperature. X-ray measurements were performed at room temperature using a Kuma KM-4 four-circle diffractometer and the graphite monochromatic CuK$\alpha$ radiation [18].

The structures were solved by heavy-atom methods with SHELXS-86 and refined by the full-matrix least-squares methods using SHELXL-93 with anisotropic parameters for all non-hydrogen atoms [19,20]. No correction for absorption has been made. The positions of all hydrogen atoms were determined from the differential Fourier synthesis [21,22]. The positions of the hydrogen atoms have not been refined. The atomic scattering factors were those of neutral atoms incorporated in SHELXL-93 [20]. Details of the measurements of the crystal data of the complexes (M-THBD) and the refinement parameters are listed in Table 2.

### Preparation of the complexes

<u>Complexes of composition $ML_2(NO_3)_2\cdot H_2O$ with M= Co(II), Ni(II) and Cu(II)</u>

To a solution of 1 mmol of appropriate metal nitrate dissolved in 5.0 ml of redistilled water, a solution of 2 mmol of THBD dissolved in 25.0 ml of hot (60-70°C) absolute ethanol was added under stirring. The solution was then left for crystallization. The precipitated complexes were filtered off, recrystallized from hot ethanol, washed successively with acetone, diethyl ether, and dried *in vacuo* at 25°C. The yields were 70-80%.

<u>Complexes of composition $ML_2Cl_2\cdot H_2O$ with M= Pd(II) and Pt(II)</u>

One mmol of $PdCl_2$ was dissolved in 5.0 ml of hot (water bath) DMF and the solution was diluted with 50.0 ml of acetone. Apart, 1 mmol of $K_2PtCl_4$ was dissolved in 5.0 ml of redistilled water. Then, to each solution, 2 mmol of THBD dissolved in 25.0 ml of hot (60-70°C) absolute ethanol were added under stirring. The solutions were left for crystallization, the complexes were filtered off, washed successively with hot ethanol, acetone and diethyl ether, and dried *in vacuo* at 25°C. The yields were 70-80%.

<u>The antiproliferative assay *in vitro*</u>

Test solutions of the compounds (1 mg/ml) were freshly prepared for each test by dissolving them either in 100 or 200 µl of DMSO + 900 or 800 µl of water *pro injectione* or, if soluble, in water only. Then the compounds were diluted in a culture medium (described below) to reach final concentrations of 100, 10, 1, 0.1 and 0.01 µg/ml. Cells of the following human cancer lines were used: A 549 (non-small cell lung carcinoma), MCF-7 and T47D (breast cancer), HCV-29 T (bladder cancer), MES-SA (uterine cancer) and HL-60 (promyelocitic leukemia). All lines were obtained from the American Type Culture Collection (Rockville, Maryland, USA) and cultured in Cell Culture Collection of the Department of Tumor Immunology, Institute of Immunology and Experimental Therapy, Wroclaw, Poland. Twenty four hours before application of the tested compounds, the cells were placed in 96-well plates (Costar, USA) at density of $10^4$ cells per well in 100 µl. The cells were cultured in an opti- MEM medium supplemented with 2 mM glutamine (Gibco, Warszawa), 50 µg/ml streptomycin (Polfa, Jelenia Gora). 50 U/ml penicillin (Polfa, Jelenia Gora) and 5% fetal calf serum (Gibco, Grand Island, USA). The cells were cultured with the agents for 72 hrs at 37°C in a humid atmosphere saturated with 5% $CO_2$.

The *in vitro* proliferation inhibition test, SRB (cell lines A-549, MCF-7, T47D, HCV-29 T and MES-SA) was applied as described in Skehan's paper [23].

<u>Proliferation inhibition test, MTT (cell line HL-60)</u>

Results of the tests were recorded by the colorimetric method from experiments carried out in flat-bottomed 96-well plates. Cells suspended in 100µl of culture medium were placed in the wells, in a number of $10^4$ per well. After 24-hr under culture conditions, successive 100 µl portions of either the neat medium (control of cell growth) or the medium containing a compound to be tested were added. The plates were incubated under identical conditions over successive 72 hrs at 37°C. After incubation, 20 µl a MTT (MTT stands for 3-(4,5-dimethylthiazol-2-yl)-2,5-diphenyltetrazolium bromide; Sigma, St. Louis, MO; stock solution: 5 mg/ml) solution was added. After a 4-hr incubation at 37°C, 80 µl of a lysing buffer was added consisting of 225 ml of dimethylformamide, 67.5 ml of sodium dodecyl sulfate (both purchased from Sigma, St. Louis, MO) and 275 ml of distilled water. After successive 24 hrs of incubation at 37°C, optical density of the liquid contained in the wells was recorded on a RM Multiskan (Labsystem, Helsinki, Finnland), at 570 nm.

Zero adjustment of the photometer was accomplished by the so-called negative control, *i.e.* by the liquid taken from the well containing the culture medium without cells. In each experiment, samples of particular concentration of the compounds were applied in triplicate. The tests were likewise carried out in triplicate.

### RESULTS AND DISCUSSION

In order to determine the composition of the coordination compounds, elemental analyses (C, H, N, metal) were run (Table 1). The results of magnetic measurements for the Co(II), Ni(II) and Cu(II) complexes of THBD match the theoretical ones, while the Pd(II) and Pt(II) compounds turned out to be diamagnetic.

**Table 1.** Analytical and physical data for the complexes.

| Complex | Colour | M.p. (°C) | Found (calc.) % | | | | μ (BM) |
|---|---|---|---|---|---|---|---|
| | | | C | H | N | M | |
| $CoL_2(NO_3)_2 \cdot H_2O$ (1) | pink | dec. 200 | 39.5 (39.8) | 2.8 (2.7) | 18.4 (18.6) | 9.6 (9.8) | 5.4 |
| $NiL_2(NO_3)_2 \cdot H_2O$ (2) | blue | dec. 205 | 39.7 (39.8) | 2.9 (2.7) | 18.3 (18.6) | 10.0 (9.7) | 2.8 |
| $CuL_2(NO_3)_2 \cdot H_2O$ (3) | green | dec. 200 | 39.3 (39.5) | 2.4 (2.6) | 18.1 (18.4) | 10.1 (10.4) | 1.8 |
| $PdL_2(Cl_2)_2 \cdot H_2O$ (4) | orange | dec.> 230 | 40.0 (40.2) | 2.5 (2.7) | 14.0 (14.1) | 17.6 (17.8) | dia. |
| $PtL_2(Cl_2)_2 \cdot H_2O$ (5) | yellow | dec.>235 | 34.7 (34.9) | 2.3 (2.3) | 12.0 (12.2) | 28.7 (28.4) | dia. |

Fig.1 shows a schematic structure of the THBD molecule together with calculated electron densities on optional electron-donating atoms. As seen, the optional electron-donating atom is the azomethine nitrogen of the imidazole ring (-0.044). However, complexation through the nitrogen atom of the thiazole ring, carrying negligible positive charge (+0.004) cannot be ruled out.

### CRYSTALLOGRAPHY

Monocrystals needed for the determination of the crystal structure could only be obtained with the Cu-THBD compound.

Details of the measurements for complex (3) crystal data together with the refinement parameters are shown in Table 2. As seen in Fig. 2, the Cu(II) ion in coordinated by lone electron pairs of the azomethine nitrogen atoms of the imidazole and thiazole rings and, in addition, by one of the oxygen atoms of the nitrate group to form a pentacoordinate structure. Such a coordination mode is in excellent agreement with the calculated electron densities (Fig.1).

Small differences in bond lenghts in the immediate surrounding of the central ion can be explained in terms of hydrogen bonds between one of the hydrogen atoms of water molecule and an oxygen atom of the coordinated nitrato group as well as between the oxygen atom of the non- coordinated nitrate group and the hydrogen atoms of the benzimidazole moiety. A chelating effect cannot be excluded, as well.

### Infrared spectroscopy

The IR spectrum (4000–400 $cm^{-1}$ range) of the complex resembles that of the non-coordinated ligand. Certain differences are noted over the stretching vibration range of the system of conjugate C=C and C=N double bonds of the compounds.

Table 3 lists absorption bands characteristic of thiabendazole and its complexes. As seen, the largest displacements due to complexation relative to thiabendazole are noted for the C=N and C=C bands.

The C-N bands are only little shifted, whereas those due to C-S remain unchanged. Remarkable are bands due to stretching and deformation vibrations of the water molecules as well as of coordinated and non-coordinated $NO_3^-$ groups [24,25] .

These are due to the bending and stretching vibrations of the metal-oxygen bonds (for the Co(II), Ni(II) and Cu(II) complexes) and of the metal-nitrogen bonds (for the Pd(II) and Pt(II) compounds). Absorption bands at 291, 296, 315, 350 and 347 $cm^{-1}$ are likely to be due to vibrations of the dative metal-nitrogen linkages for the complexes of Co(II), Ni(II), Cu(II), Pd(II) and Pt(II), whereas those at 437, 437 and

438 $cm^{-1}$ are due to M-O linkages in the complexes of Co(II), Ni(II) and Cu(II), respectively, with thiabendazole [26,27].

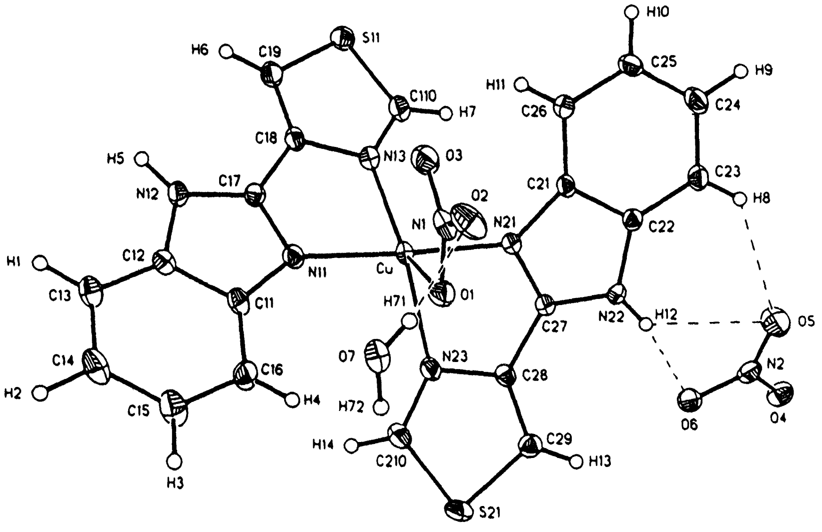

Fig.2. Molecular structure and atom labeling scheme for complex (3).

**Table 2.** Crystal data and structure refinement for $Cu(THBD)_2(NO_3)_2$ $H_2O$

| Empirical formula | $C_{20}$ $H_{16}$ Cu $N_8$ $0_7$ $S_2$ |
|---|---|
| Formula weight | 608.07 |
| Temperature | 293 (2) K |
| Wavelength | 0. 71073 Å |
| Crystal system | monoclinic |
| Space group | P $2_1$/c |
| Unit cell dimensions | a = 7.924 (2) Å<br>b = 27.022 (5) Å<br>c = 11.275 (2) Å<br>β = 103.87 (3)° |
| Volume | 2343.8 (8) $Å^3$ |
| Z | 4 |
| Density (calculated) | 1.72 $Mg/m^3$ |
| Absorption coefficient | 1.172 $mm^{-1}$ |
| F(000) | 1236 |
| Crystal size | 0.15 x 0.15 x 0.20 mm |
| 2 θ range | 3.5 – 27.0° |
| Range of h, k, l | -7/10, -34/32, -14/13 |
| Reflection collected/ unique | 15203 / 5046 [R (int)= 0.0208] |
| Refinement method | Full- matrix least squares on $F^2$ |
| Data[I> 2σ (I)]/Parameters | 4451 / 407 |
| Goodness-of-fit on $F^2$ | 1.06 |
| Final R indices [I>2σ (I)] | $R_1$= 0.037, w $R_2$= 0.086 |
| R indices (all data) | $R_1$= 0.042, w $R_2$= 0.089 |
| Largest diff. Peak and hole | 1. 74 and –0.87 e. $Å^{-3}$ |
| Weighting scheme | $w=1/[\sigma^2(F_o^2)+ (0.05P)^2+ 0.00P]$ where $P= (F_o^2+ 2F_c^2)/3$ |
| Diffractometer | Kuma KM-4 |
| Programs used | SHELXL-93, SHELXL-86 |
| Deposition number | CCDC167906 |

Selected most important bond length and angles are as follows: Cu-N(21)-1.960(2), Cu-N(11)-1.973(2), Cu-N(13)-2.077(2), Cu-O(1)- 2.2226(19) Å, N(21)-Cu-N(11)-176.67(8), N(21)-Cu-N(13)-95.53(8), N(11)-Cu-

N(13)-81.66(8), N(21)-Cu-N(23)-80.01(8), N(11)-Cu-N(23)-103.21(8), N(13)-Cu- N(23)-133.89(8), N(21)-Cu-O(1)-89.18(8), N(11)-Cu-O(1)-91.94(8), N(13)-Cu- O(1)- 141.57(7), N(23)-Cu-O(1)-84.51(7)°.

On the basis of these findings, it can be speculated that the copper(II) compound of THBD has a pentacoordinate structure. A similar structure can be assigned to the cobalt(II) and nickel(II) complexes, whilst those of palladium(II) and platinum(II) form planar square structures.

**Table 3.** Characteristic frequencies in $cm^{-1}$ of the ligand and its complexes.

| Compound | $\nu H_2O$ | $H_2O$ | C=N | C=C | C-N | C-S | $NO_3^-{}_{coord.}$ | $NO_3^-{}_{ncoord.}$ |
|---|---|---|---|---|---|---|---|---|
| Ligand | ------ | ------ | 1580 | 1420 | 1245 | 750 | ---- | -------- |
| 1 (Co) | 3400 | 1650 | 1620 | 1435 | 1248 | 750 | 1335 | 1380 |
| 2 (Ni) | 3400 | 1650 | 1600 | 1440 | 1247 | 750 | 1330 | 1390 |
| 3 (Cu) | 3500 | 1630 | 1560 | 1400 | 1246 | 750 | 1330 | 1420 |
| 4 (Pd) | 3500 | 1625 | 1600 | 1440 | 1250 | 750 | ---- | -------- |
| 5 (Pt) | 3500 | 1630 | 1600 | 1430 | 1250 | 750 | ---- | -------- |

Antiproliferative assay

Results of the assay, presented as $ID_{50}$ (this denotes a dose of a compound inducing the proliferation inhibition of 50 per cent of the cancer cell population), carried out for cells of particular cancer cell lines, are shown in Table 4.

The adopted activity criterion for the compounds in the *in vitro* screening was an $ID_{50}$ level not exceeding 4 μg/ml [28]. No compound satisfied that criterion.

As seen in Table 4, only the Pt(II)-THBD complex exhibited activity matching that set by the criterion in an assay against the HL-60 leukemia cells and those of urinary bladder carcinoma HCV-29 T cells.

**Table 4.** *In vivo* cytotoxic activity of the thiabendazole complexes against human cancer cell lines.

| Compound | Cell line/$ID_{50}$ [μg/ml] | | | | | |
|---|---|---|---|---|---|---|
| | **T47D** | **MCF7** | **MES-SA** | **HL-60** | **A549** | **HCV-29T** |
| Pd THBD | *neg* | *neg* | *neg* | 61.0 ± 2.0 | *neg* | *neg* |
| Co THBD | 61.2 ± 1.3 | *neg* | 87.7 ± 1.1 | 23.2 ± 1.0 | ------------ | ---------- |
| Cu THBD | 30.7 ± 1.1 | 34.5 ± 1.0 | 34.5 ± 1.0 | 24.6 ± 1.2 | ------------ | ---------- |
| Ni THBD | *neg* | *neg* | *neg* | 56.0 ± 2.3 | ------------ | ---------- |
| Pt THBD | 29.0 ± 1.3 | 39.2 ± 1.2 | 37.8 ± 1.0 | 5.8 ± 1.6 | 49.0 ± 1.0 | 11.0 ± 4.0 |
| Cisplatin | 2.1 ± 1.8 | 3.2 ± 1.2 | 1.4 ± 1.0 | 0.2 ± 1.5 | 4.0 ± 1.0 | 0.7 ± 0.01 |

Of the cancer cells tested, only the leukemia cells have been found to be sensitive to all the coordination compounds tested. Besides, the copper(II) and platinum(II) complexes of THBD exhibited the ability to inhibit proliferation of cells of all the cancer lines tested. Again, the Co(II) – THBD compound inhibited proliferation of some cell lines only, notably those of T47D, MES-SA and HL-60, whereas the palladium(II) and nickel(II) complexes exhibited only a low activity against the HL-60 cells.

To sum up, as far as the *in vivo* antiproliferative activity assay conducted is concerned, it can be concluded that further studied should be focused on the HL-60 leukemia cells.

### REFERENCES

1. M.M. Jones, *J. Chem. Educ.*, **53**, 342 (1976)
2. M.M. Jones, *Inorg. Chim. Acta.*, **107**, 235 (1985)
3. A. Bult, Metal complexes of sulfanilamides in pharmaceutical analysis and therapy. Copyright by Marcel Dekker, Inc., New York and Basel, 261-276 (1983)
4. B. Rosenberg, L. van Camp, J.E. Trosko, V.H. Mensour, *Nature*, **222**, 385 (1969)
5. B. Rosenberg, *Cancer*, **55**, 2303 (1985)
6. K. Maskos, *Wiad. Chem.*, **35**, 735 (1988)
7. J.L. Van der Veer, J. Reedijk, *Chem. Brit.*, 775 (1988)

8. W. Kaim, B. Schwederski, Inorganic elements in the chemistry of life. John Wiley and Sons, Chichester, New York, Brisbane, Toronto, Singapore, 363 (1964)
9. U.P. Singh, S. Signh, S.M. Singh, *Metal Based Drugs*, **5**, 35 (1998)
10. P. Yang, M. Guo, *Metal Based Drugs*, **5**, 41 (1998)
11. B.K. Keppler, D. Wehe, H. Endres, W. Rupp, *Inorg. Chem.* **26**, 844, 4366 (1987)
12. F. Coletta, R. Ettore, A. Gambaro, *J. Inorg. Nucl. Chem.* **37**,314 (1975)
13. C. G. van Kralingen, J. Reedijk, *Inorg. Chim. Acta.*, **30**, 171 (1978)
14. C. G. van Kralingen, J.K. Ridder, J. Reedijk, Inorg. Chim. Acta, **36**, 69 (1979)
15. M.M. Muir, G.M. Gomez, M.E. Cadiz, J.A. Muir, *Inorg. Chim, Acta*, **168**, 47 (1990)
16. M.Z. Wisniewski, J. Wietrzyk, A. Opolski, *Archiv. Immuno. Ther. Exp.* **48**, 51 (2000)
17. M.Z. Wisniewski, W. J. Surga, B. Lenarcik, *Trans. Met. Chem.*, **15**, 63 (1990)
18. Kuma KM-4 Software User Guide, Version 3.1., Kuma Diffraction, Wroclaw, Poland, 1976
19. G.M. Sheldrick, *Acta Cryst., Sect. A.*, **46**, 467 (1990)
20. G.M. Sheldrick SHELXL-93. Program for the Refinement of Crystal Structure, University of Göttingen, Geramany (1993)
21. W. Wolodkiewicz, W. Brzyska, T. Glowiak, *Polish J. Chem.* **70**, 409 (1996)
22. M.Z. Wisniewski, Glowiak T., *Polish J. Chem.*, **72**, 514 (1998)
23. P. Skehan, R. Storeng, D. Scudiero, A. Hanks, J. Mc Mahon, D. Visitica, J. Waren, H. Bokesch, S. Kenny, M. Bogol, *J. Natl. Cancer Inst.*, **82**, 1107 (1990)
24. F. Akhtar, F. Hug, A.C. Skapski, *J. Chem. Soc. Dalton*, 1353 (1972)
25. M.Z. Wisniewski, K. Kurdziel, M. Scendo, *Polish J. Chem.*, in press
26. M. Gabryszewski, B. Wieczorek, Spectr. Letters, **33**, 23 (2000)
27. M. Gabryszewski, B. Wieczorek, *Polish J. Chem.*, **73**, 2061 (1999)
28. R.S. So, A.A. Adjei, *J. Clin. Oncol.*, **17**, 409 (1999)
29. J. Reedijk., *Chem. Commum.*, 801 (1996)
30. R. J. Geran, N.H. Greenberg, M.M. Mc Donald, A.M. Schumacher, B.J. Abbott, *Cancer Chemother. Rep.*, **3**, 59 (1972)

**Received: September 13, 2001 – Accepted: October 10, 2001 – Accepted in publishable format: November 15, 2001**

